# Multiscale optoacoustic assessment of skin microvascular reactivity in carotid artery disease

**DOI:** 10.1016/j.pacs.2024.100660

**Published:** 2024-10-30

**Authors:** Angelos Karlas, Nikoletta Katsouli, Nikolina-Alexia Fasoula, Mario Reidl, Rhiannon Lees, Lan Zang, Maria del Pilar Ortega Carrillo, Stefan Saicic, Christoph Schäffer, Leontios Hadjileontiadis, Daniela Branzan, Vasilis Ntziachristos, Hans-Henning Eckstein, Michael Kallmayer

**Affiliations:** a Chair of Biological Imaging at the Central Institute for Translational Cancer Research (TranslaTUM), School of Medicine and Health, Technical University of Munich, Germany; b Institute of Biological and Medical Imaging, Helmholtz Zentrum München, Neuherberg, Germany; c Clinic and Polyclinic for Vascular and Endovascular Surgery, TUM University Hospital, Hospital rechts der Isar, Technical University of Munich, Munich, Germany; d Chair for Computer Aided Medical Procedures & Augmented Reality, Technical University of Munich, Munich, Germany; e DZHK (German Centre for Cardiovascular Research), partner Site Munich Heart Alliance, Munich, Germany; f Department of Biomedical Engineering & Biotechnology, Khalifa University of Science and Technology, Abu Dhabi, UAE; g Department of Electrical and Computer Engineering, Aristotle University of Thessaloniki, Thessaloniki, Greece

**Keywords:** Microvascular endothelial dysfunction, Raster-scan optoacoustic mesoscopy, Microvascular features, Cardiovascular disease, Non-invasive imaging

## Abstract

Microvascular endothelial dysfunction may provide insights into systemic diseases, such as carotid artery disease. Raster-scan optoacoustic mesoscopy (RSOM) can produce images of skin microvasculature during endothelial dysfunction challenges via numerous microvascular features. Herein, RSOM was employed to image the microvasculature of 26 subjects (13 patients with single carotid artery disease, 13 healthy participants) to assess the dynamics of 18 microvascular features at three scales of detail, i.e., the micro- (<100 μm), meso- (≈100–1000 μm) and macroscale (<1000 μm), during post-occlusive reactive hyperemia challenges. The proposed analysis identified a subgroup of 9 features as the most relevant to carotid artery disease because they achieved the most efficient classification (AUC of 0.93) between the two groups in the first minute of hyperemia (sensitivity/specificity: 0.92/0.85). This approach provides a non-invasive solution to microvasculature quantification in carotid artery disease, a main form of cardiovascular disease, and further highlights the possible link between systemic disease and microvascular dysfunction.

## 1 Introduction

Carotid artery disease is a frequent cause of ischemic stroke, the incidence of which has been increasing worldwide [1]. Various image-based and serum biomarkers have been proposed to further understand disease pathophysiology and possibly improve disease management [2], [3], [4]. Ultrasound (US) imaging is the first-line technique used in carotid artery disease, not only for the detection of the stenosis but also when deciding to proceed with surgery (quantification of >70 % stenosis is an indication for surgery). Other relevant US-based biomarkers, such as the intima-media thickness (IMT) or the presence of ulcerations in the common carotid artery (CCA), have also been employed as sentinel biomarkers of general cardiovascular health [3], [5]. Furthermore, serum biomarkers, such as inflammatory, lipid-related, hematologic, and metabolic biomarkers, have been proposed as indirect indicators of carotid artery disease [2], [6]. While serum analysis is often inconvenient for the patient, it remains an easily accessible tool; however, the lack of relevant clinical evidence precludes the routine use of this analysis in the management of carotid artery disease. Therefore, there is still a need to investigate novel biomarkers in patients with carotid artery disease.

Exploring endothelial dysfunction (ED), a reporter of general cardiovascular health and a surrogate marker of carotid atherosclerosis, could lead to the identification of such biomarkers [7], [8]. ED may be investigated in different vascular beds (e.g., microvascular or macrovascular), potentially offering insights into biomarkers linked to carotid artery disease and atherosclerotic cardiovascular disease (CVD) in general [9]. Recent studies highlight the importance of accurate microvascular ED assessment in the management of CVD or carotid artery disease [10]. For example, evidence suggests that microvascular ED not only precedes but also predicts the development of macrovascular atherosclerosis, the most important cause of CVD [11].

Peripheral tissues, such as the skin, offer a representative vascular bed that is easily accessible and can be non-invasively tested with several techniques. As skin microvascular ED is affected by CVD, its detailed characterization is of great interest, as shown in relevant studies [9]. For example, it has been shown that microvascular ED examined using peripheral arterial tonometry (PAT) of the finger is a predictor of adverse cardiovascular events, such as myocardial infarction [12]. In a cohort of patients suffering from different CVDs, including carotid artery disease, coronary artery disease (CAD), and peripheral arterial disease (PAD), it has been shown that even patients with a single vascular disease (of the three abovementioned types) displayed significantly decreased PAT-extracted microvascular ED in comparison to patients without vascular disease [13]. However, to date, there has been no corresponding analysis for carotid artery disease alone. Furthermore, although PAT is a non-invasive and easy-to-use technique to assess microvascular ED, it does not offer direct imaging of the examined microvasculature, instead showing rough volumetric changes of the blood within the measured tissues [12]. In other words, PAT extracts a one-dimensional signal that could correspond to many different tissues, such as the skin, subcutaneous fat, nerves, and blood vessels, without direct imaging of the microvasculature and its changes. Given this, several aspects of microvascular ED that could lead to the identification of novel biomarkers for single carotid artery disease remain unexplored.

In this work, we employ raster-scan optoacoustic mesoscopy (RSOM), a novel non-invasive skin imaging technique, to perform a thorough analysis of microvascular ED in patients with single carotid artery disease, i.e., without any other form of atherosclerotic CVD. The use of optoacoustics has been applied in imaging various aspects of carotid artery disease and its complications, as well as in the assessment of ED, in both clinical and preclinical settings [14], [15], [16], [17], [18]. Specifically, RSOM has been shown to not only offer detailed visualizations of the skin, but also to facilitate the identification of disease-specific biomarkers in the dermal microvasculature. For example, studies show that RSOM analyses of skin microvasculature allow the assessment of disease stage in patients with diabetes [19], [20].

Herein, we extend this excellent capability of shifting from purely static images to dynamic ED tests, with the aim of identifying new RSOM-based biomarkers that may be linked to single asymptomatic extracranial carotid artery disease. For simplicity, the term “single carotid artery disease” is employed to denote “single asymptomatic extracranial carotid artery disease”. After extracting 18 features from the dermis layer, we investigate their fluctuation over time during post-occlusive reactive hyperemia (PORH) tests of the microvascular ED. Following these observations of feature dynamics, we categorize features into three scales of detail, i.e., micro- (<100μm), meso- (≈100–1000μm), and macroscale (>1000μm), introducing a novel multiscale approach to microvascular ED assessment. Prior research indicates that the “mesoscale” features appear to be most affected (deteriorated) in patients with diabetes [19]. Finally, we identify the subgroup of features that are most relevant to carotid artery disease by determining which features best classify healthy volunteers and patients with single carotid artery disease. Our proposed solution allows us to employ this simple, fast, and non-invasive imaging technology to examine novel biomarkers with possible links to CVD and carotid artery disease.

## 2 Methods

### 2.1 Participant characteristics and preparation

The study was approved by the ethics committee of the Technical University of Munich (Protocol #326/19S). The demographics and clinical characteristics of the recruited participants are provided in Table 1. In total, 13 patients with single extracranial carotid artery disease (without other major atherosclerotic disease, such as CAD or PAD) and 13 age-matched healthy participants were included in this study.

**Table 1:** Demographics and clinical characteristics of study participants.

	N	Age	Sex	BMI	Hypertension	Smoking	Hyperlipidemia	Diabetes
Patients	13	70.2±9.0	9 M/4 F	26.0±2.7	10	4	10	2
Healthy	13	64.5±10.5	5 M/8 F	25.3±4.0	4	0	2	0

N: Number of participants, Age in years (mean ± standard deviation), M: Male, F: Female, BMI: Body mass index in kg/m^2^ (mean ± standard deviation)

After giving detailed informed consent, recruited participants were placed in a quiet, semi-dark examination room with normal room temperature (≈23°C) and relaxed in a relaxed sitting position for 15 min before the beginning of the examination. Participants were asked to avoid exercise for 24 h and fast for 6 h prior to the examination. During the PORH test, each participant was placed in a seated position with the dominant arm lying on an examination table at the level of the heart. To prevent the effects of melanin, skin areas with high melanin content were excluded from the scanning process.

### 2.2 Post-occlusive reactive hyperemia test

**Fig. 1:**
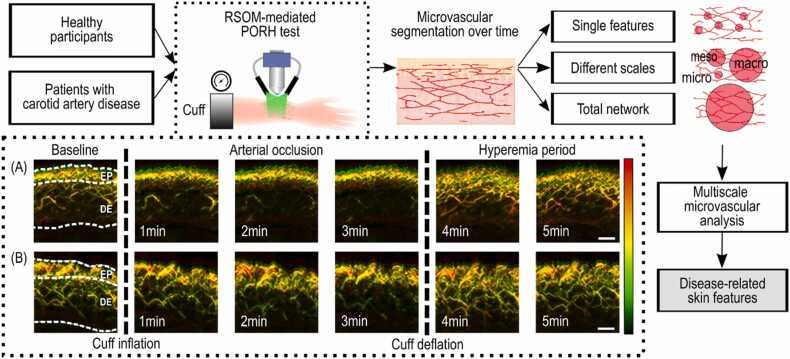
**: Workflow of the current study, from participant inclusion to identification of disease-related features.** Exemplary series of raster-scan optoacoustic mesoscopy (RSOM) images are shown, taken from (A) a healthy participant and (B) a patient with carotid artery disease during a post-occlusive reactive hyperemia (PORH) test. EP: Epidermis, DE: Dermis. The colormap indicates the size of the microvessels: red – larger vessels, green – smaller vessels. Only microvessels in the volumetric DE are analyzed. Scale bar: 500 µm.

A fully deflated blood pressure cuff (sphygmomanometer) of appropriate size was placed around the patient’s upper arm on the dominant side. The blood pressure of the participant was measured three times, and the average systolic (SBP) and diastolic (DBP) blood pressures were registered as the participant’s SBP/DBP. We then positioned the RSOM measurement probe on the volar side of the forearm, approximately 10 cm distally from the elbow joint, after covering the skin with a dedicated, transparent plastic thin foil. After ensuring perfect contact between the RSOM probe and the skin, we conducted the RSOM measurements of skin microvascular ED as described below. A detailed description of our workflow, along with an exemplary image series of a healthy participant and patient with carotid artery disease, is depicted in Fig. 1. As observed, the vascular network, especially in the region of the dermoepidermal junction, is far richer in the healthy volunteer (Fig. 1A) during the hyperemia period. Furthermore, the vessels within the dermis are larger (higher levels of red color representing lower frequencies) in the healthy volunteer images corresponding to the hyperemia period compared to the patient (Fig. 1B) recordings.

First, a baseline image of the skin microvasculature was recorded. A single RSOM image recording with the RSOM device takes ≈45 s. Next, we inflated the cuff at a pressure of 40 mmHg more than the registered SBP of the participant to ensure occlusion of the brachial artery. Palpation of the brachial artery distally to the cuff confirmed that the brachial artery was occluded. The cuff remained fully inflated for three minutes (avascularization period) while three more RSOM images were recorded. At the end of the 3-minute avascularization period, the cuff was abruptly fully deflated, allowing for the reperfusion of the arm. Finally, the skin microvasculature was imaged for another two minutes (hyperemia period). Thus, for each measurement, six images of the skin microvasculature across three phases (baseline, arterial occlusion, hyperemia period) were recorded in total.

### 2.3 Optoacoustic imaging with RSOM

A commercial RSOM system (RSOM C50, iThera Medical GmbH, Munich, Germany) was employed to record all RSOM data used in this study. RSOM technology has been thoroughly described previously [20]. Briefly, a green light pulsed laser (532 nm) illuminated the examined skin region with laser pulses at a rate of 350 Hz. The laser beam was moved by means of two mechanical stages to scan an area of 4x2mm^2^ skin region in approximately 45 s. Ultrasound waves generated upon light absorption were detected by means of an ultrasound detector with a bandwidth of 10–120 MHz (central frequency: 50 MHz). Then, the US signals recorded during each scan were motion-corrected and reconstructed into a tomographic volume of the skin using a dedicated reconstruction algorithm. The reconstructed data were then “colorized” with a green-yellow-red colormap, where the green color corresponds to the high frequencies (40–120 MHz, smaller microvessels) and the red color to the low frequencies (10–40 MHz, larger microvessels). The yellow color results from the combination of green and red voxels/pixels where both smaller and larger microvessels appear. For each minute of the PORH test, one RSOM image was recorded, providing a total of six volumetric images for each test/participant. We then characterized the microvascular network for each volume as described below.

### 2.4 Extraction of dermal features

To characterize the structure of the skin microvascular network visualized in RSOM volumes, we segmented the dermis (DE), the vascularized layer of the skin, and its microvasculature. Our previously described data analysis method is described in the supplementary Fig.S1 [19]. After automatically segmenting the DE and its microvasculature, we extracted 18 morphological microvascular features that provide a representative description of the dermal microvascular network [19]. The following 18 dermal features initially extracted for each RSOM volume are (Table 2): 1. Average angle of junctions, 2. Average length of the vessels, 3. Average vessel tortuosity, 4. Ratio of vessel length-to-width, 5. Average vessel diameter, 6. Average vessel volume, 7. Number of vessels, 8. Density of junctions, 9. Density of vessels (Vascular density), 10. Ratio of junctions-to-vessels, 11. Number of junction-to-junction branches, 12. Number of junction-to-endpoint branches, 13. Number of junctions, 14. Thickness of the DE layer, 15. Total area of vessels, 16. Average intensity of the optoacoustic signal within the vessels, 17. Average intensity of the optoacoustic signal in the DE layer, 18. Area of the DE layer. Following initial extraction, the microvascular features were further categorized based on the scale of detail represented, as described in Table 2 below.

**Table 2:** Initially extracted microvascular dermal features and their scale of detail.

### 2.5 Scale analysis of the extracted dermal features

The extracted dermal features for each RSOM image were further categorized into three classes representing different scales of detail: the “microscale”, which includes features referring to single-vessels parameters, such as the average vessel diameter or volume; the “mesoscale”, which contains features describing the local organization of the microvascular network, such as the number of vessels or density of junctions); and the “macroscale”, which includes rough features of the structure of the entire dermal layer, such as the thickness or the average optoacoustic signal within the DE layer. The scale of detail for each calculated feature is also given in Table 2. By exploring three different scales of detail, we aimed to provide a complete description of the changes within the cutaneous microvascular network during dynamic tests of microvascular reactivity and endothelial function.

### 2.6 Dynamic multiscale assessment of the microvascular network

**Fig. 2:**
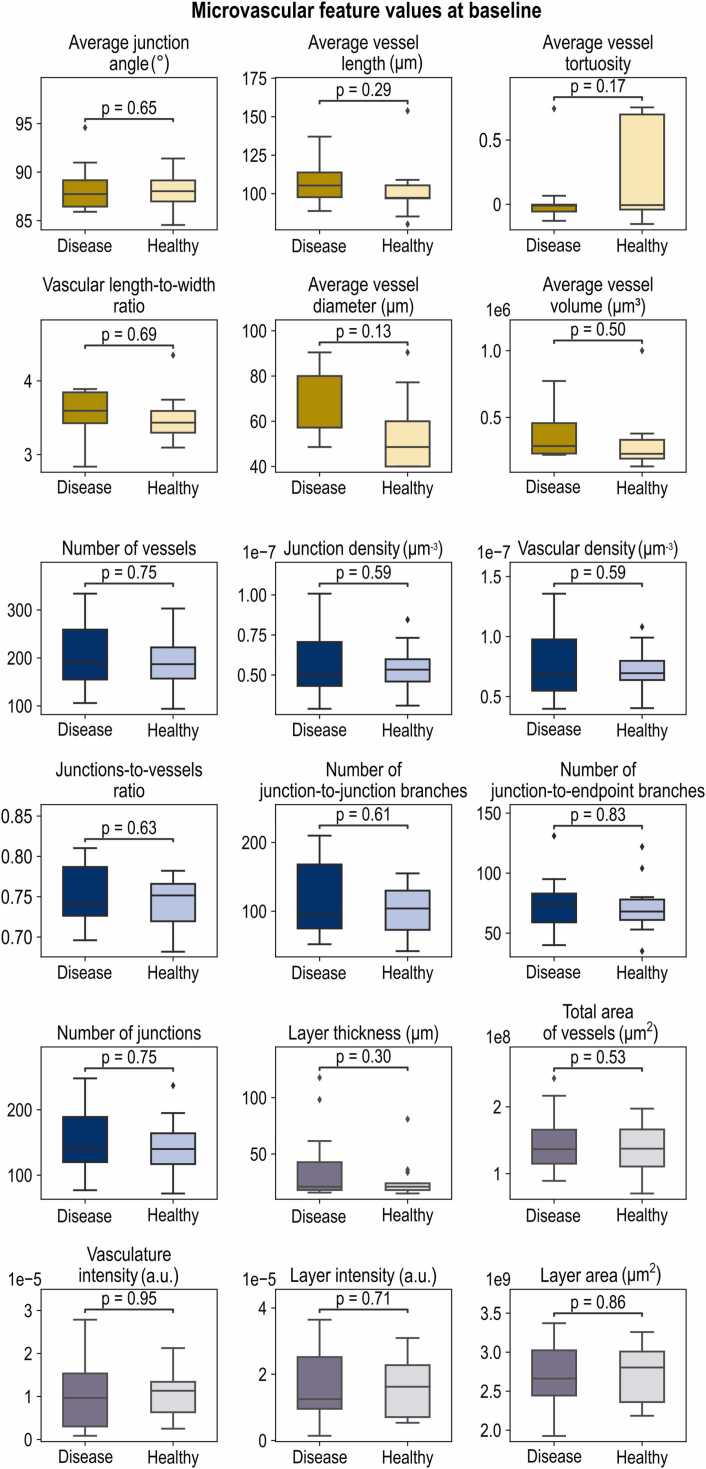
**Statistical analysis of the 18 RSOM-extracted microvascular features in the two examined groups (13 healthy volunteers and 13 patients with single carotid artery disease) at the baseline.** Microscale features are marked in yellow, mesoscale features are marked in blue, and macroscale features are marked in gray. None of the features is characterized by a statistically significant difference between the two groups, p>0.05. A two-sided Student’s t-test was used to calculate all p-values. The boxplot’s center line represents the median value, while the box limits represent the first and third quartiles. The whiskers, representing the minima and maxima, extend to 1.5 times the interquartile range.

**Fig. 3:**
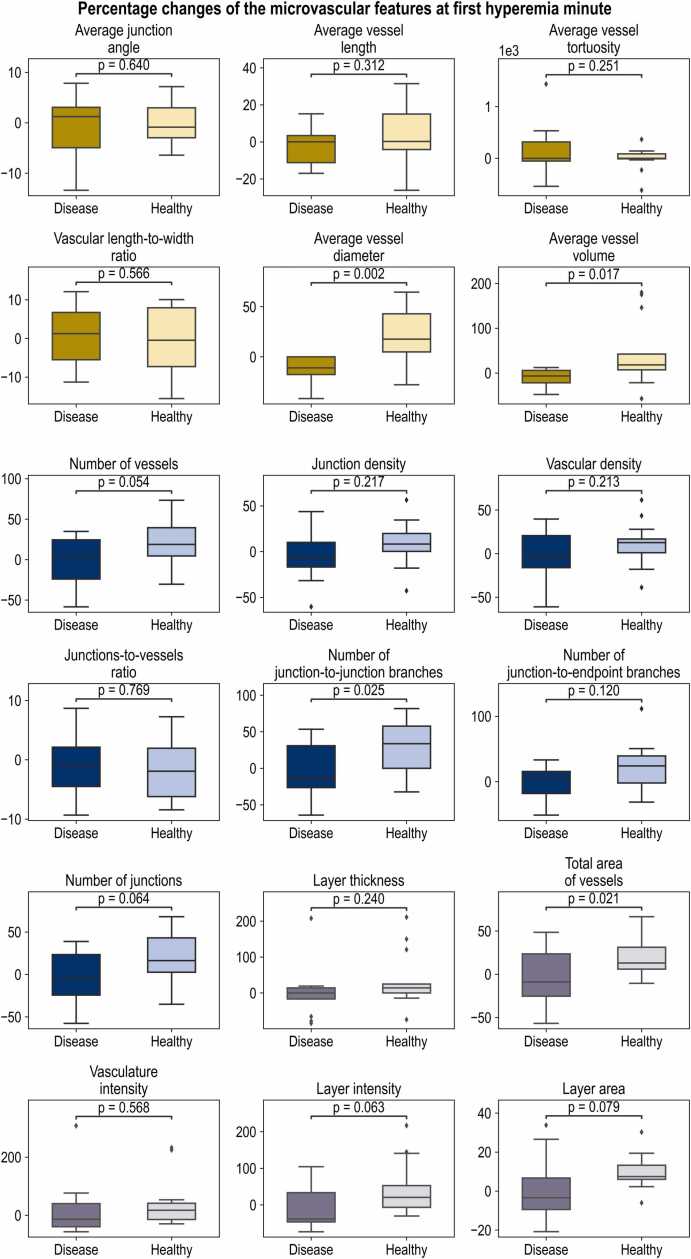
**Statistical analysis of the percentage changes of the 18 RSOM-extracted features in the two examined groups (13 healthy volunteers and 13 patients with single carotid artery disease) at one minute after cuff deflation.** Microscale features are marked in yellow, mesoscale features are marked in blue, and macroscale features are marked in purple. A two-sided Student’s t-test was used to calculate all p-values. The boxplot’s center line represents the median value, while the box limits represent the first and third quartiles. The whiskers, representing the minima and maxima, extend to 1.5 times the interquartile range.

**Fig. 4:**
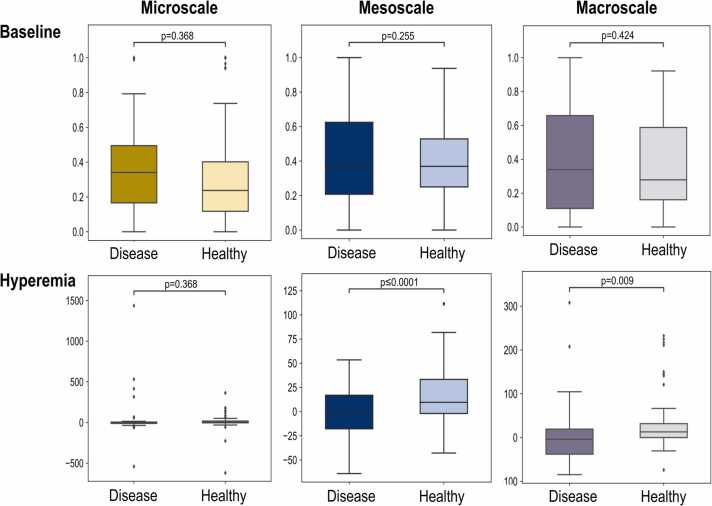
**Statistical analysis of the features between the two examined groups (13 healthy volunteers and 13 patients with single carotid artery disease) per scale of detail.** Upper row: normalized feature values at the baseline (normalization per feature), and lower row: percentage change of the features at one minute after cuff deflation. The microscale is marked in yellow, the mesoscale is marked in blue, and the macroscale is marked in gray. A two-sided Student’s t-test was used to calculate all p-values. The boxplot’s center line represents the median value, while the box limits represent the first and third quartiles. The whiskers, representing the minima and maxima, extend to 1.5 times the interquartile range.

After extracting the 18 dermal features from all recorded images (six volumetric images per participant, one for each minute of the PORH test), we calculated the percentage changes of these features over time compared to the baseline RSOM image from each participant. For each feature, we calculated five values that described the dynamics of the feature during the PORH test. We assessed the dynamics of each feature separately (Fig. 2, Fig. 3) and also performed an analysis of the changes occurring for each scale of detail: the micro-, meso- and macroscale (Fig. 4).

To identify the skin microvascular features that might be more relevant to carotid artery disease, we performed a classification step between the two examined groups (healthy participants and patients with carotid artery disease) for each minute of the PORH test. Before applying a random forest (RF) classifier, we reduced the group of the 18 initially extracted features by means of the “SelectKBest” feature selection algorithm [21]. Following the dimensionality reduction, the algorithm selected the most “disease-relevant” features and used them as input for the RF model. The RF produced a new feature-based index for each minute of the PORH test. The resulting “disease-relevant” feature-based index was further used to classify the two groups. To implement this methodology, a leave-one-subject-out (LOSO) approach was employed. The area under the receiving operator curve (AUC) served as a descriptor of efficacy for the index-based classifier on a per-minute basis. This approach identified a subgroup of the 18 initially extracted dermal features that led to the best AUC for each minute of the PORH test or the most “disease-relevant” features that led to the best differentiation from the control group.

With this step, we not only managed to identify the dermal features most relevant to the disease (i.e., the subgroup of features that achieved the highest AUC) but also to provide a holistic description of the microvasculature dynamics over time by using the entire set of the 18 initially extracted features which span all scales of detail. Based on the outcomes described in the next section, the best classification was achieved one minute after the deflation of the cuff, as expected according to physiology, which shows that the vascular reactivity one minute after the cuff deflation (compared to the baseline) is descriptive of the endothelial functional state of the examined individual [22].

### 2.7 Confounding analysis

To validate whether specific characteristics of the examined participant groups might affect the feature-based classification performance, we performed a confounding analysis. We investigated the following parameters: age, sex, body mass index (BMI), smoking habit, and the presence of hypertension, hyperlipidemia and diabetes. The analysis aimed to identify possible confounders and included a comparison between three classification models (generalized linear models, GLMs): a first model based only on the corresponding confounder (e.g., age), a second model based on the confounder and the produced feature-based index, and a third model based on the confounder, the feature-based index and an interaction parameter between the index and confounder. The classification performance for each model was finally estimated via both the confounding residuals and the p-value achieved between the two groups: patients with carotid artery disease and healthy participants.

In addition, after evaluating the results of the GLMs, we conducted a multivariate linear regression analysis, including all potential confounding parameters and the feature-based index as independent variables to further investigate the impact of the parameters on our results [23]. Since only 2 out of 26 participants have diabetes and only 4 out of 26 have hyperlipidemia, there is limited data for these variables. The lack of statistical significance for the interaction term (p≈1) in the third model suggests that these two variables do not influence the relationship between the feature-based index and the disease. As a result, they were not included in the multivariate linear regression analysis.

## 3 Results

In this work, we provide a thorough analysis of the skin microvascular dynamics during PORH tests in patients with single carotid artery disease. Over time, we calculated and followed 18 dermal features extracted from RSOM images. As a first step, we investigate the dynamics of each one of the 18 RSOM-extracted dermal features and their differences between the two groups: the healthy participants and the patients with single carotid artery disease. In Fig. 2, we present the differences between all 18 features at the baseline, i.e., before conducting the PORH test. Features are categorized based on their scale of detail, with the microscale features presented in yellow, mesoscale features in blue, and macroscale features in gray. Our analysis shows that, at the baseline, none of the initially extracted features were characterized by statistically significant differences between the two investigated groups. Such an observation highlights the need to explore microvascular endothelial dysfunction and its significance as a marker of carotid artery disease.

In Fig. 3, we present the percentage change of each RSOM-extracted feature compared to the baseline one minute after cuff deflation, when we expected to see the most descriptive ED hyperemic state. Several features showed a statistically significant difference (p<0.05) between the healthy participants and the patients with single carotid artery disease. In the microscale, the average vessel diameter and volume were significantly reduced in the patient group (p=0.002 and p=0.017, respectively) one minute after the cuff deflation. In the mesoscale, a relative decrease in the number of junction-to-junction branches was seen during hyperemic reaction (p=0.025). Correspondingly, in the macroscale, the total area of vessels was significantly (p=0.021) decreased in the patient group compared to the healthy group at one minute into the hyperemia period. In Fig. S2-S5 (*see* Supplementary material), we display the percentage changes of each RSOM-extracted feature compared to the baseline. The measurements are taken at one minute after cuff inflation, two minutes after cuff inflation, three minutes after cuff inflation, and two minutes after cuff deflation, respectively.

As a next step, we relied on the multiscale categorization of the extracted features to explore whether different dynamic profiles of microvascular reactivity characterize each scale of microanatomic detail. Thus, we provide a scale-based analysis of the extracted microvascular features. As observed in Fig. 4, the meso- and the macroscales demonstrated significant differences between the two groups at one minute after cuff deflation (hyperemia period). As expected, none of the scales shows significant differences at the baseline.

We explored if a specific subgroup of the extracted features is more relevant to single carotid artery disease. As observed in Table 3, the best classification performance, as shown by the calculated AUC, is achieved at one minute after cuff deflation. Furthermore, for this time point, 9 out of 18 dermal features are selected because they provide the best classification and are, thus, considered to be the most “relevant” biomarkers of single carotid artery disease. Concerning the classification within one minute after cuff inflation, the model could not classify because almost no differences in the feature values between this minute and baseline were observed. The selected features used for the calculation of the “disease-relevant” feature-based index and their relevant importance for the calculation of the index are provided in Table 4. The subgroup of the most “disease-relevant” features includes features from all scales of detail.

**Table 3:** Analysis of feature-based classification performance over time.

Parameter	Minutes after the start of the PORH test				
	Arterial occlusion			Hyperemia period	
	1 min	2 min	3 min	4 min	5 min
Number of selected features	N/A	6	7	**9**	11
AUC	N/A	0.54	0.62	**0.93**	0.76
Accuracy	N/A	0.62	0.65	**0.88**	0.73
Sensitivity	N/A	0.46	0.31	**0.92**	0.85
Specificity	N/A	0.77	1.00	**0.85**	0.62
Positive Predictive Value	N/A	0.67	1.00	**0.86**	0.69
Negative Predictive Value	N/A	0.59	0.59	**0.92**	0.80

**Table 4:** The 9 features with the highest relevance to disease, their scale of detail, and relative importance.

Finally, our confounding analysis (see Methods 2.7) showed that none of the possible confounders affected our model’s ability to classify participants into two groups (diseased or healthy). In other words, for all potential confounders (age, sex, BMI, smoking habit, the presence of hypertension or hyperlipidemia), the second model based on the confounder and the produced feature-based index led to the best classification performance compared to the other two models. In fact, the confounding residuals were decreased in the case of the second model compared to the first one (based only on the corresponding confounder). Furthermore, the p-value achieved by means of the second model was significantly better than the p-value achieved by the third model based on the confounder, the feature-based index, and an interaction parameter between the index and the corresponding confounder. The confounding analysis results are provided in Table 5 (upper panel).

**Table 5:** Confounding analysis results.

**Generalized Linear Models**						
Confounder	Model 1		Model 2		Model 3	
	Residuals	p-value	Residuals	p-value	Residuals	p-value
Age	33.734	N/A	13.816	≤ 0.00001	11.885	0.165
Sex	33.526	N/A	15.279	≤ 0.0001	12.635	0.104
BMI	35.390	N/A	16.580	≤ 0.0001	13.398	0.075
Hypertension	30.248	N/A	12.979	≤ 0.0001	8.697	0.039
Smoking habit	29.767	N/A	17.261	≤ 0.001	17.261	0.999
Hyperlipidemia	28.091	N/A	9.615	≤ 0.0001	6.807	0.094
Diabetes	33.104	N/A	13.999	≤ 0.0001	13.999	1.000
Model 1: Confounder Model 2: Confounder and feature-based index Model 3: Confounder, feature-based index, and their interaction						

**Multivariate Linear Regression Model**			
Variable	Regression Coefficient	Standard error	p-value
Age	−0.0029	0.003	0.304
Sex	−0.1332	0.130	0.318
Body mass index (BMI)	−0.0120	0.135	0.930
Hypertension	0.2475	0.140	0.092
Hyperlipidemia	0.2522	0.138	0.083
Feature-based index	0.9438	0.221	≤ 0.001

Furthermore, based on the multivariate linear regression analysis (Table 5 lower panel), the feature-based index is the only variable showing statistical significance (p≤0.05) with the highest regression coefficient, indicating its importance as the most significant variable.

## 4 Discussion and conclusions

With the rates of carotid atherosclerosis rising worldwide [24], the pursuit of novel disease-related biomarkers or surrogate markers of CVD in general is imperative. Studies have shown that the skin is well-suited for measurements to detect such biomarkers. For example, the dermal accumulation of advanced glycation end products (AGEs) and the related increase in skin autofluorescence (AF) have been suggested as markers of atherosclerotic CVD and, particularly, carotid atherosclerosis [25], [26].

In this study, we focused on the skin microvascular ED, a known barometer for general cardiovascular health that seems to precede macrovascular ED and has been associated with carotid atherosclerosis. More specifically, we present for the first time a detailed RSOM-based multiscale analysis of dermal microvascular features during PORH tests. We also identify specific disease-related features in patients with single carotid artery disease. By examining patients with single carotid artery disease (without CAD or PAD), we minimize the possible effect of such conditions on the peripheral skin microvasculature, for example, in subjects with reduced heart ejection fraction or peripheral arterial pathology in the arm: both conditions that could affect peripheral skin microvasculature.

Skin microvascular ED has previously been studied in patients with carotid artery disease of the skin during PORH tests using PAT [13]. However, PAT and other available techniques employed to assess skin microvascular dysfunction do not provide in-depth insights into detailed features of the skin microvasculature. On the contrary, the current study employs RSOM to thoroughly analyze the skin microvasculature, opening up new possibilities for exploring endothelial dysfunction in CVD. Our approach may well provide the clinician with an additional non-invasive tool for future patient management.

Furthermore, the applied multiscale approach enables the investigation of different aspects of the skin microvascular ED. The main finding that the meso- and macroscale – or else the scales describing the microvascular organization and the rough skin characteristics – are primarily affected in carotid artery disease shows that not all aspects of the dermal vasculature are affected the same. Such an observation further highlights the role of the skin as an exciting target for the future definition of disease-relevant markers in CVD and other diseases.

Our study is limited by the relatively small size of our cohort, preventing easy generalization of our observations. However, the identification and recruitment of subjects with only a single carotid artery disease is challenging, as carotid artery disease is strongly associated with both CAD and PAD. Nonetheless, this study and future studies are needed to validate RSOM, an innovative technology, which has not yet been disseminated and, thus, is not available in the majority of healthcare units. Such studies on RSOM would further accelerate the clinical translation of this promising technology and lead to its widespread use.

In conclusion, the study aims to explore possible new RSOM-based biomarkers associated with carotid artery disease. Even if the current approach does not focus explicitly on patients with vulnerable or "high-risk" carotid plaques, it may provide preliminary insights into defining markers associated with plaque vulnerability in the future. Especially since we still need improved preventive approaches for carotid-associated stroke and carotid plaque vulnerability has been associated with stroke independently of the stenosis grade [27]. Of course, more focused and thorough studies are needed to be conducted to shift the paradigm from being based purely on the grade of stenosis towards a more holistic approach and better patient management.

## CRediT authorship contribution statement

**Angelos Karlas:** Writing – original draft, Visualization, Methodology, Investigation, Funding acquisition, Formal analysis, Data curation, Conceptualization. **Nikoletta Katsouli:** Writing – original draft, Visualization, Software, Methodology, Formal analysis, Data curation. **Nikolina-Alexia Fasoula:** Writing – original draft, Visualization, Methodology, Data curation. **Mario Reidl:** Writing – original draft, Data curation. **Rhiannon Lees:** Writing – original draft. **Lan Zang:** Writing – original draft. **Maria del Pilar Ortega Carillo:** Writing – original draft. **Stefan Saicic:** Writing – original draft. **Christoph Schäffer:** Writing – original draft. **Leontios Hadjileontiadis:** Writing – review & editing, Methodology. **Daniela Branzan:** Supervision. **Vasilis Ntziachristos:** Writing – original draft, Supervision, Funding acquisition. **Hans-Henning Eckstein:** Supervision, Resources. **Michael Kallmayer:** Writing – review & editing, Supervision, Resources.

## Declaration of Competing Interest

The authors declare the following financial interests/personal relationships which may be considered as potential competing interests: Vasilis Ntziachristos reports a relationship with sThesis GmbH that includes: equity or stocks. Vasilis Ntziachristos reports a relationship with iThera Medical GmbH that includes: equity or stocks. Vasilis Ntziachristos reports a relationship with I3 Inc that includes: equity or stocks. Vasilis Ntziachristos reports a relationship with Spear UG that includes: equity or stocks. All other authors declare that they have no known competing financial interests or personal relationships that could have appeared to influence the work reported in this paper.

## Data Availability

Data will be made available on request.

## Footnotes

Appendix A Supplementary data associated with this article can be found in the online version at doi:10.1016/j.pacs.2024.100660.
