# Supplementary material for: Multiscale optoacoustic assessment of skin microvascular reactivity in carotid artery disease
Source: Photoacoustics. 2024 Oct 30;40:100660. doi: 10.1016/j.pacs.2024.100660 (PMC11624498; doi:10.1016/j.pacs.2024.100660)
Supplement: Supplementary file 1 — Supplementary material [file mmc1.docx]

**Supplementary material**


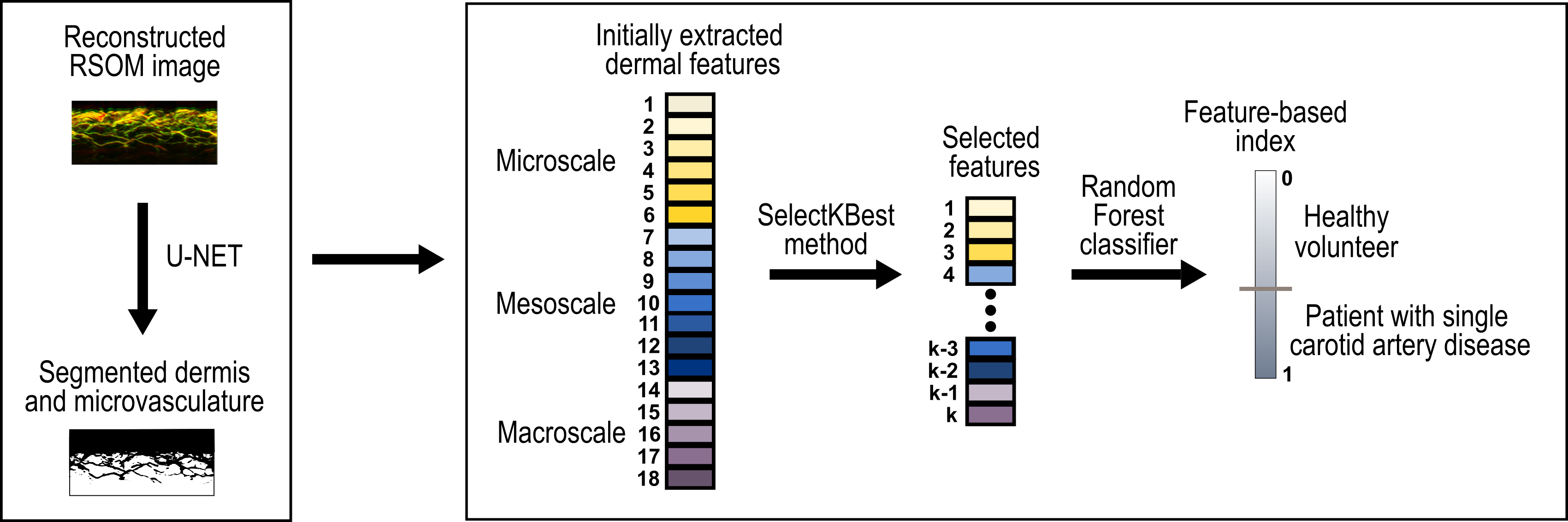


**Fig. S1. Detailed description of the data analysis algorithm [14]**. The algorithm was applied for each minute of the endothelial dysfunction (post-occlusive hyperemia, PORH) test.


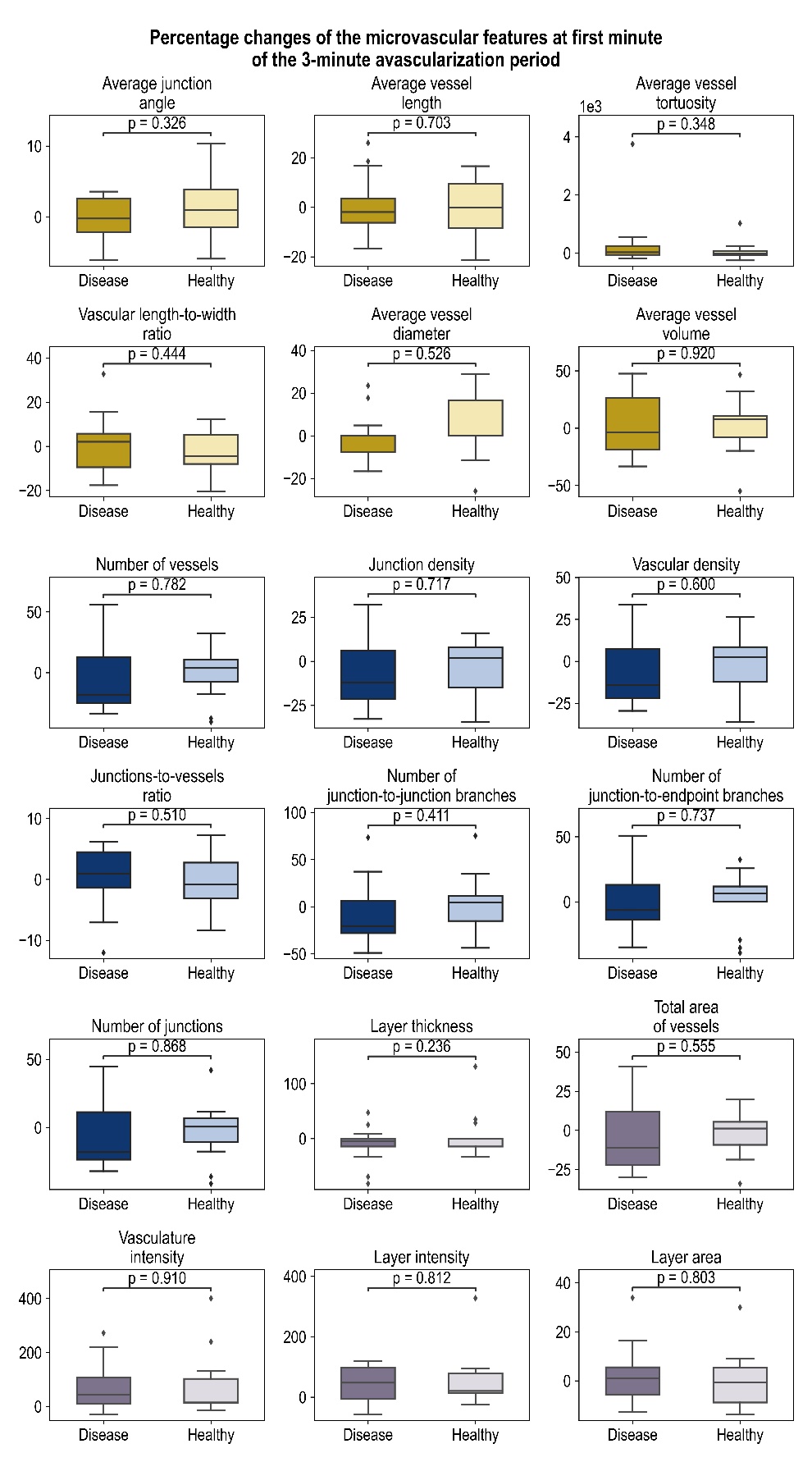


**Fig. S2. Statistical analysis of the percentage changes of the 18 RSOM-extracted features in the two examined groups (13 healthy volunteers and 13 patients with single carotid artery disease) at one minute after cuff inflation.** Microscale features are marked in yellow, mesoscale features are marked in blue, and macroscale features are marked in purple. A two-sided Student’s t-test was used to calculate all p-values. The boxplot’s center line represents the median value, while the box limits represent the first and third quartiles. The whiskers, representing the minima and maxima, extend to 1.5 times the interquartile range.


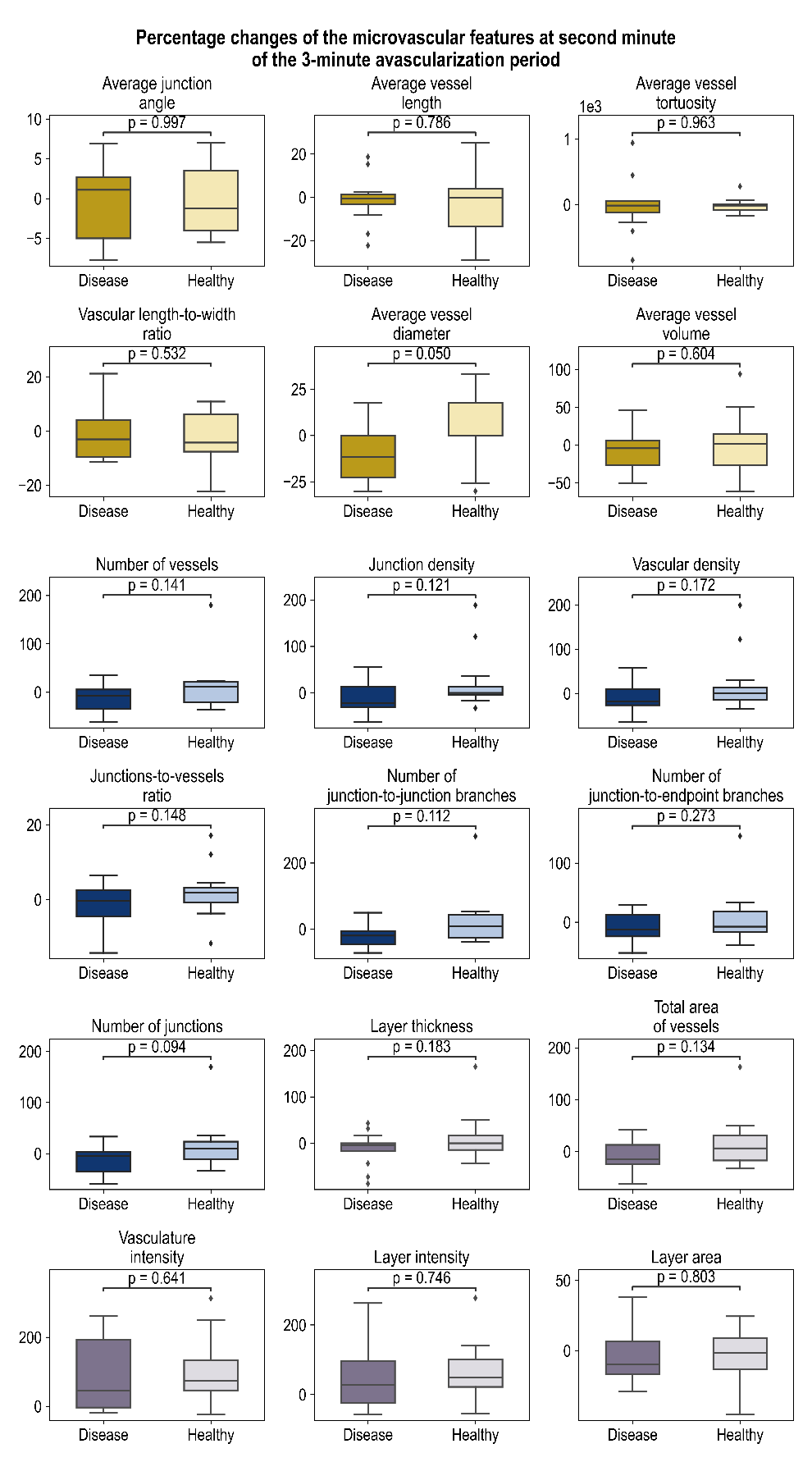


**Fig. S3. Statistical analysis of the percentage changes of the 18 RSOM-extracted features in the two examined groups (13 healthy volunteers and 13 patients with single carotid artery disease) at second minute after cuff inflation.** Microscale features are marked in yellow, mesoscale features are marked in blue, and macroscale features are marked in purple. A two-sided Student’s t-test was used to calculate all p-values. The boxplot’s center line represents the median value, while the box limits represent the first and third quartiles. The whiskers, representing the minima and maxima, extend to 1.5 times the interquartile range.


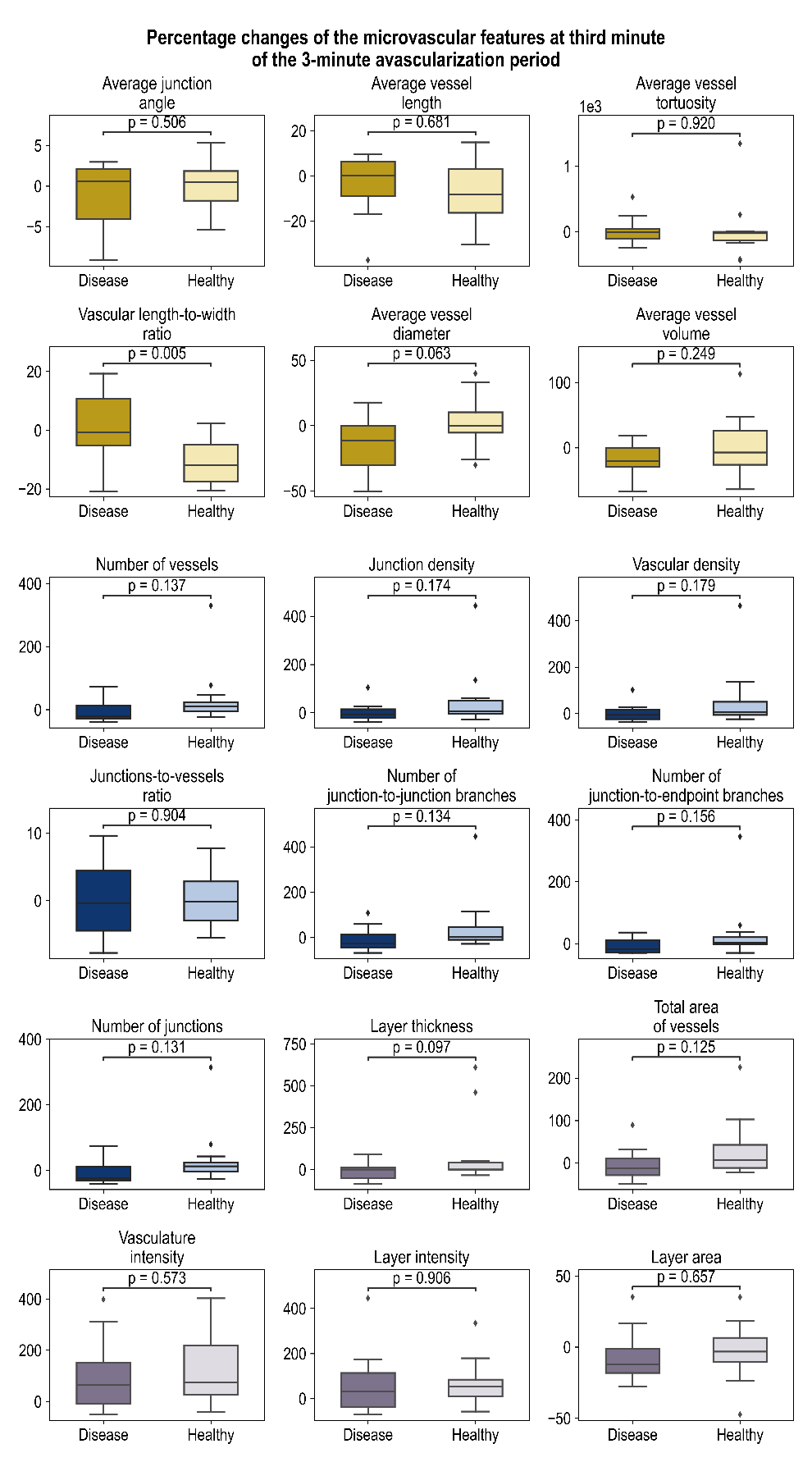


**Fig. S4. Statistical analysis of the percentage changes of the 18 RSOM-extracted features in the two examined groups (13 healthy volunteers and 13 patients with single carotid artery disease) at third minute after cuff inflation.** Microscale features are marked in yellow, mesoscale features are marked in blue, and macroscale features are marked in purple. A two-sided Student’s t-test was used to calculate all p-values. The boxplot’s center line represents the median value, while the box limits represent the first and third quartiles. The whiskers, representing the minima and maxima, extend to 1.5 times the interquartile range.


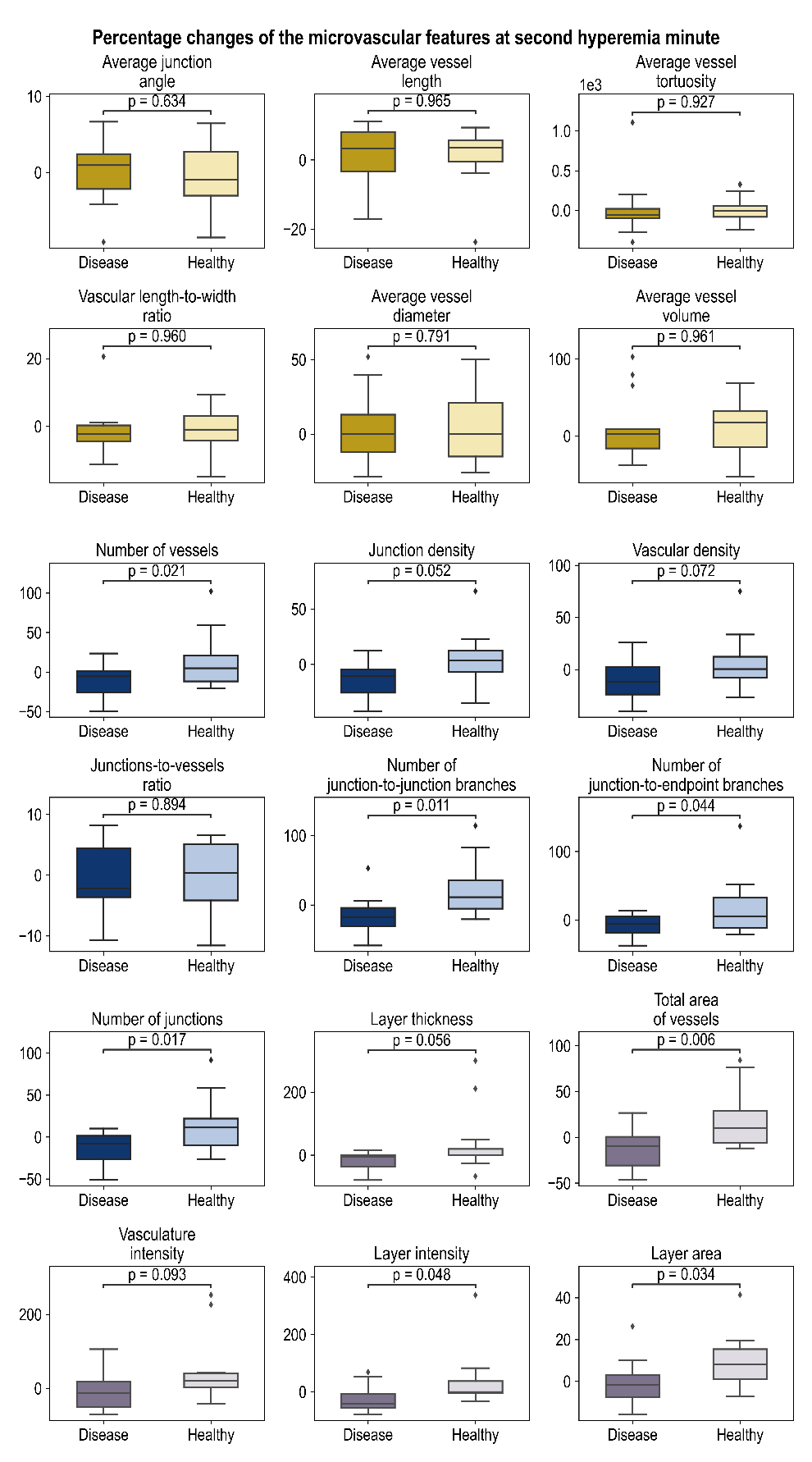


**Fig. S5. Statistical analysis of the percentage changes of the 18 RSOM-extracted features in the two examined groups (13 healthy volunteers and 13 patients with single carotid artery disease) at second minute after cuff deflation.** Microscale features are marked in yellow, mesoscale features are marked in blue, and macroscale features are marked in purple. A two-sided Student’s t-test was used to calculate all p-values. The boxplot’s center line represents the median value, while the box limits represent the first and third quartiles. The whiskers, representing the minima and maxima, extend to 1.5 times the interquartile range.
